# The Medical Association of Georgia

**Published:** 1867-03

**Authors:** 


### EDITORIAL AND MISCELLANEOUS

### THE MEDICAL ASSOCIATION OF GEORGIA.

The annual meeting of the Medical Association of Georgia will convene in the city of Griffin, on Wednesday, the 12th of April. A large attendance of the profession at the approaching session is greatly to be desired, as questions vital to the future usefulness of this body, and to the pecuniary interests of the physicians, particularly of certain sections of the State, will be presented for consideration. The present position of former master and slave, especially in the cotton growing region of the State, has resulted in great embarrassment and pecuniary loss to the physician. Their attendance is still required upon the *freedman*, but in quite the majority of cases, without any assurance of remuneration. The question then naturally arises, what can the physician do to protect himself? This is an important matter, and one that is exciting considerable discussion in the former slave States. As an evidence of the embarrassment felt by the profession, and the necessity of some concert of action, we will here give some extracts from a letter received a short time since, from two intelligent and prominent physicians of the State:

"Believing your views on the questions about to be propounded, will have weight and influence with our medical community, before whom they will be brought for action, we take the liberty of thus addressing you, and requesting your opinion, as follows:

"1st. Is there anything contrary to the letter or spirit of the code of medical ethics, as adopted by the American Medical Association, or by the Medical Association of our

State, in offering to make contracts with corporations, plantations, or other bodies of operatives? Before proceeding further, we would say, there has never been any objection, we believe, to a physician's contracting with a corporation, town, or city, to do the practice of such body for a given sum. But heretofore, when negroes were slaves, and therefore property, the master was considered sufficiently interested in the well-being of that property to furnish them medical attention on his own account. Then it was generally agreed among physicians that making contracts was derogatory to the dignity of the profession, and injurious to its interests. Now, however, that the negro has ceased to be property, there are three points of practical interest bearing on the question. *The former master's* interest in the health of the negro has ceased, except to a very limited extent. *The negro*, as a paid laborer, receives wages insufficient to buy his clothing, meet his inconsiderate expenses, and pay in addition, his doctor's bill, from the beginning to the end of the year. If the negro dies before the expiration of the second or third month, after a long illness, the employer has no assets in his hand to pay for his medical attention. At the end of the year it will often be found that the negro has drawn his wages up to date, and has nothing left to pay his doctor's bill. These are not supposed cases; they have occurred to us in several instances. This subject was brought before the Medical Society of this town, in January, 1866, and it was thought best not to change the established custom in this particular, but to hold the planter personally responsible for any bill contracted on his plantation, and a notice to that effect was published in the newspaper. The result of the last year's experience has shown the entire insufficiency of this plan. In but few instances were physicians able to collect their bills, and, in many cases, lost them altogether, the planters declaring that the negroes were in their debt, etc. On the other hand, planters are willing and anxious, at the beginning of the year to make contracts for their freedmen, and assess each one his *pro rata* for the doctor's bill. Under such an arrangement, they are willing to become personally responsible for the amount of the contract. The negroes, too, are generally willing, and anxious to make an arrangement by which they are assured of medical attention during the year, for a sum easily within their power to pay, no matter how much sickness they have in their family. It seems to us that the experience of last year should teach the physician the importance of making some arrangement by which he will certainly receive some compensation for his services, instead of risking his whole year's labor for nothing. We ask, then, in view of the above facts:

"2d. Supposing the system of contracting be contrary to the letter or spirit of the code of medical ethics,, would not any physician, or physicians be justifiable in disregarding it in that particular?"

While there is nothing in the letter or spirit of the code of ethics of the American Medical Association, which, if we mistake not, has, without change, been adopted by the Medical Association of Georgia, against contracts of any kind, still the great body of the profession has ever, and we think justly, condemned any system of contracting, other than that of a Federal, State, or municipal character.

But the great struggle for our independence, through which we have just passed, has resulted in a complete revolution of our social and political status. We must meet promptly the requirements of our changed condition, and in doing that, should adopt such a course that will result in the greatest good to all. The freedman still requires the attention of the physician. It is greatly to the interest of the planter that the laborer receive prompt and efficient medical attention. The physician, upon his part, must have some guarantee of remuneration, else he will be forced to leave his location, or abandon his profession. This will certainly be the result in those rich cotton districts, where, perhaps, three-fourths of the population are *freedmen*. The plan to be pursued in this changed condition, for the protection of the profession, is a question upon which there appears to be quite a diversity of opinion. The Southern Medical and Surgical Journal is decidedly in favor of the *contract* system. It says: "It is plain that the *contract* system is the only one now practicable, for general purposes, and it behooves the profession to adopt some uniform scale of charges which will secure general support. If the physicians of each neighborhood are left to such desultory plans as each may see proper to adopt, the result will be continued dissatisfaction to all parties. If a uniform system prevails, each county will soon be mapped out into practicing districts, included in a radius of five or eight miles, thereby, securing a fair distribution of the labor and gain, and equalizing incomes. We have been in communication with physicians in different parts of the State, and find a general testimony borne to the value and practicability of the contract system, the terms being fixed by the distance and number of laborers."

The Richmond Medical Journal objects to the contract system, and offers the opinion that the mass of the profession would, at present, as strenuously oppose the contract system as heretofore. In lieu of this plan of adjustment, this valuable Journal suggests the following as likely to give the requisite protection to all interested.

"The following plan will, it is believed, be entirely satisfactory to all interested. In all sections of country, the employers of laborers are enabled, either by experience, or inquiry, to determine what, in a series of years, would be the average yearly cost of medical attendance, for a given number of employees. Of course the sum would vary proportionately with the latitudinal and local causes of disease, with the character of the laborer, etc. There would, however, be no difficulty in determining the probable sum necessary at each locality. This amount being approximately fixed for the year or month, it would be then necessary to provide for its payment, by a *pro rata* weekly or monthly deduction from the wages of all employed. This aggregate deduction would constitute a fund for the payment of the medical attendant or attendants; each employee being, justly, allowed to decide the important matter for himself, and to select the physician most acceptable to himself or family. Each practitioner should present his bill for service actually rendered, this bill being strictly in accordance with the provisions of the appropriate city, village, or county fee-bill. At the close of each month, any unexpected balance of the fund should either be refunded to the employee or placed to his credit, for the next month, or quarter, as may be mutually acceptable. If the fund be insufficient, it should be temporarily increased. For securing entire satisfaction, the accounts in connection with this fund, should, by agreement, be subject to the inspection of all interested."

This would certainly be the most equitable adjustment of the difficulty; and, if it could be carried out in good faith, would be the very best plan. But we have our fears of its practicability.

We candidly admit that we are not yet satisfied as to the best plan of adjustment. There are three parties concerned; and we must, as far as practicable, make it satisfactory to all interested. If nothing better than the contract system is found practicable, for the protection of the practitioner, we would certainly favor that plan of adjustment.

But, as there is some conflict of opinion, and as all, we feel confident, are willing to do for the best, it is desirable that the profession, at least, in the sections of the State most interested, confer with each other, and with the planters, or contractors with whom they are in daily contact, and by delegates, or in mass, meet the profession at the approaching session of the Association, prepared to give all the information, that some action may be had which will for ever settle this vexed question.

By the following resolution, adopted at the last meeting of the Association, it will be seen that, at the approaching session, action, in regard to a change of one of the prominent features of the body, will be had:

"*Resolved*, That the permanent location of the Association at some suitable place, in the opinion of this meeting, is called for by its highest interests; and that in view of said interests, we do invite and call upon its members in every portion of the State, to meet with us at our next annual meeting, and settle definitely this question."

We feel that this is an important question, and one that will have much to do with the future usefulness of the Association. Before action is taken, either for or against the permanent location of this organization, it is desirable that the question be canvassed in the auxiliary societies of the State, and where they do not exist, among the individual members of the profession, so that delegates and members may come prepared to represent the wishes of the profession.

One other question of equal importance to the above, was before the Association for action at its last meeting, the consideration of which was deferred until the approaching session.

We have reference to the proposed change of the constitution in regard to membership. The constitution, as it now stands, permits to membership licentiates in good standing in the profession. The change proposed is to exclude all, without any reference to scientific attainment, standing in the profession, or other qualifications, from membership in the Association, unless they have a diploma from some accredited medical school.

As a number of the members of the Association, and heretofore, some of the most zealous and scientific, belong to that class that it is hereafter proposed to exclude, and as the change proposed would necessarily require a change in the letter and spirit of the code of ethics of the American Medical Association, as adopted by this Association, we hope the change will not be made. There are many reasons, in our opinion, why this feature of the constitution should not be changed; but, as it is simply our object to call the attention of the profession to the contemplated action, we will not pursue the subject further.

We would again urge the great importance, both to the Association and to the profession, of a full attendance. Let none stay away for the reason that they are not members, as all physicians in good standing will, at once, be admitted to membership, and a participation in the deliberations of the Association.

